# Response to Mazori et al, “Overlap of generalized morphea and eosinophilic fasciitis after recreational exposure to epoxy resin”

**DOI:** 10.1016/j.jdcr.2025.05.001

**Published:** 2025-05-26

**Authors:** Noah Hewitt, Alicia Zbehlik

**Affiliations:** a Department of Medicine, West Virginia University School of Medicine, Morgantown, West Virginia; b Division of Rheumatology, Department of Medicine, West Virginia University School of Medicine, Morgantown, West Virginia

**Keywords:** dermatopathology, eosinophilic fasciitis, eosinophils, epoxy resin, morphea, scleroderma

*To the Editor:* We thank Mazori et al for their interest in our article and for building upon its discussion. The authors acknowledge the clinical challenge of distinguishing eosinophilic fasciitis (EF) from deep morphea. They provide helpful insights into differentiating the 2 conditions and explain why this distinction is important for the purpose of monoclonal gammopathy screening in patients with EF.^1^Additionally, they emphasize the importance of recognizing the clinical nuances between EF/morphea and systemic sclerosis, highlighting the significant therapeutic and prognostic implications.^1^ In our patient, groove sign became apparent after initial presentation (Fig 1). Notably, our patient lacked Raynaud's phenomenon, distal digital puffiness/induration, and extracutaneous manifestations of systemic sclerosis in addition to the lack of dilated nailfold capillaries mentioned in our article. We are grateful for the authors' contribution to the ongoing discussion of these complex conditions, which require careful consideration in diagnosis and treatment.

**Fig 1:**
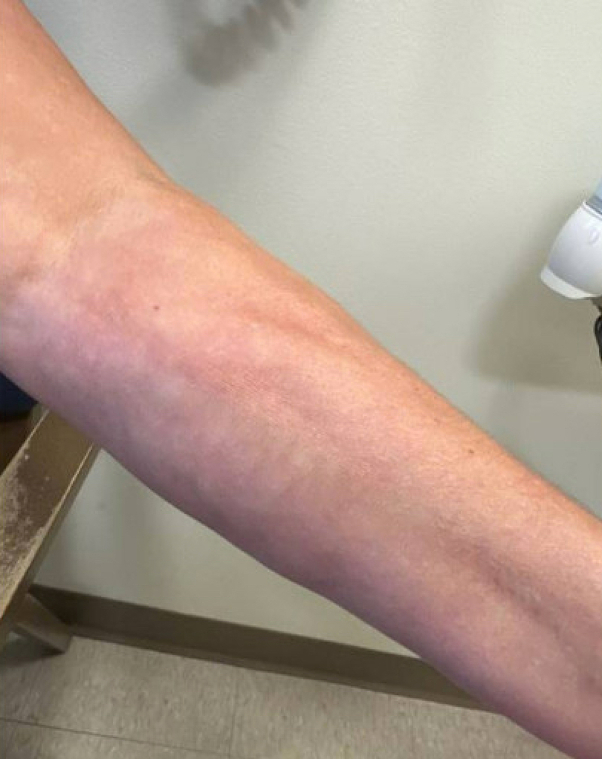
Groove sign of the left upper extremity.

## Conflicts of interest

None disclosed.
